# Transcriptome Analysis Reveals Candidate Lignin-Related Genes and Transcription Factors during Fruit Development in Pomelo (*Citrus maxima*)

**DOI:** 10.3390/genes13050845

**Published:** 2022-05-09

**Authors:** Xiaoting Li, Hantang Huang, Hafiz Muhammad Rizwan, Naiyu Wang, Jingyi Jiang, Wenqin She, Guohua Zheng, Heli Pan, Zhixiong Guo, Dongming Pan, Tengfei Pan

**Affiliations:** 1 College of Horticulture, Fujian Agriculture and Forestry University, Fuzhou 350002, China; 2170305003@fafu.edu.cn (X.L.); chrizwan51@gmail.com (H.M.R.); 1200305016@fafu.edu.cn (N.W.); jingyiJiang@fafu.edu.cn (J.J.); 000q020040@fafu.edu.cn (W.S.); fafuzgh@126.com (G.Z.); panheli@fafu.edu.cn (H.P.); gzhhs@fafu.edu.cn (Z.G.); tfpan@fafu.edu.cn (T.P.); 2 College of Horticulture, China Agricultural University, Beijing 100083, China; B20203170823@cau.edu.cn

**Keywords:** pomelo, granulation, lignin, WGCNA, transcriptome

## Abstract

Juice sac granulation (a physiological disorder) leads to large postharvest losses of pomelo (*Citrus maxima*). Previous studies have shown that juice sac granulation is closely related to lignin accumulation, while the molecular mechanisms underlying this disorder remain elusive in pomelo. Our results showed that the lignin content in NC (near the core) and FC (far away from the core) juice sacs overall increased from 157 DPA (days post anthesis) to 212 DPA and reached a maximum at 212 DPA. Additionally, the lignin content of NC juice sacs was higher than that of FC juice sacs. In this study, we used transcriptome-based weighted gene co-expression network analysis (WGCNA) to address how lignin formation in NC and FC juice sacs is generated during the development of pomelo. After data assembly and bioinformatic analysis, we found a most correlated module (black module) to the lignin content, then we used the 11 DEGs in this module as hub genes for lignin biosynthesis. Among these DEGs, *PAL* (phenylalanine ammonia lyase), *HCT* (hydroxycinnamoyl-CoA shikimate/quinate hydroxycinnamoyl transferase), *4CL2* (4-coumarate: CoA ligase), *C4H* (cinnamate 4-hydroxylase), *C3′H* (*p*-coumarate 3-hydroxylase), and *CCoAOMT1* (caffeoyl CoA 3-Omethyltransferase) were the most distinct DEGs in granulated juice sacs. Co-expression analysis revealed that the expression patterns of several transcription factors such as MYB, NAC, OFP6, and bHLH130 are highly correlated with lignin formation. In addition, the expression patterns of the DEGs related to lignin biosynthesis and transcription factors were validated by qRT-PCR, and the results were highly concordant with the RNA-seq results. These results would be beneficial for further studies on the molecular mechanism of lignin accumulation in pomelo juice sacs and would help with citrus breeding.

## 1. Introduction

‘Sanhongmiyou’ (‘SH’) [*C*. *maxima* (Burm.) Merr.] is a bud mutant of ‘Guanximiyou’ [*C. maxima* (Burm.) Merr.], which is a leading tree-fruit crop and is mainly cultivated in Fujian province of China [1]. It is a distinctive citrus fruit characterized by its thick peel, large size, and high vitamin C content [2]. It is favoured for its health-benefiting compounds, including flavonoids [3,4], volatile compounds [5], alkaloids [6], and carotenoids [7,8,9]. However, granulation is a physiological disorder among citrus species, especially in pomelo fruits, such as ‘Hongroumiyou’ (HR) [*C. maxima* (Burm.) Merr.] and ‘SH’, wherein juice sacs become abnormally hardened, shrunken, and dry with little free juice [10,11]. This disorder results in reduced nutritive qualities and commodity values, and growers also suffer heavy losses [12,13].

Previous studies have demonstrated that lignin metabolism is well correlated with the symptoms of juice sac granulation and that lignin content increases with the aggravation of citrus granulation [1]. The increase in the lignin content of juice sacs plays a significant role in the citrus fruit granulation process [2,14]. Lignin, which normally accumulates in secondary cell walls, is one of the most important components of the plant cell wall [15]. Previous evidence has shown that granulation due to excessive lignin accumulation can greatly damage fruit sensory quality during the post-harvest process [1], such as loquat (*Eriobotrya japonica*) [16,17], pear (*Pyrus bretschneideri*) [18,19], and sweet orange (*Citrus sinensis*) [20].

In plants, the lignin biosynthesis pathway involves a series of enzymes that belong to a collection of genes and gene families, including PAL (phenylalanine ammonia lyase), C4H (cinnamate 4-hydroxylase), 4CL (4-coumarate: CoA ligase), HCT (hydroxycinnamoyl-CoA shikimate/quinate hydroxycinnamoyl transferase), C3H (*p*-coumarate 3-hydroxylase), CCoAOMT (caffeoyl CoA 3-Omethyltransferase), CCR (cinnamoyl CoA reductase), COMT (caffeic acid O-methyltransferase), CAD (cinnamylalcohol dehydrogenase), and F5H (ferulate 5-hydroxylase). Some of these genes have also been isolated and characterized in pomelo [21,22]. Under the common catalysis of these enzymes, plants synthesize three different types of lignin monomers, namely, *p*-hydroxyphenyl (H) monomers, guaiacyl (G) monomers, and syringyl (S) monomers, which are then polymerized by laccases (LACs) or peroxidases [23,24,25]. The granulated juice sacs of pomelo are mainly composed of G lignin [1].

Transcription factors (TFs) that regulate lignin biosynthesis genes have been identified in a variety of plant species, including NAC [no apical meristem (NAM), *Arabidopsis* transcription activation factor (ATAF1/2), cup-shaped cotyledon (CUC2)], MYB (myeloblastosis) [25,26], and ERF (ethylene-responsive binding element) transcription factors (TFs) [17]. In *Arabidopsis* (*Arabidopsis thaliana*), the NAC TF, *SND1,* and its close homologues *NST1*, *NST2*, *VND6*, and *VND7* act as master switches that lead to the activation of the biosynthetic genes for cellulose, xylan, and lignin [27,28,29,30,31,32,33,34,35]. Zhong et al. [33] found that the simultaneous knockout of *SND1* and *NST1* completely blocked secondary wall thickening and lignin deposition in fibers. In loquat, fruit accumulating lignin during postharvest low-temperature storage, an NAC transcription factor, *EjNAC1*, was found to regulate loquat fruit lignification [36]. Subsequently, Ge et al. [37] found that *EjNAC3* is a direct regulator of loquat chilling-induced lignification via the regulation of *EjCAD*-like. While *EjAP2-1* controls interactions between the *EjMYB1* and *EjMYB2* proteins to regulate lignin biosynthesis in loquat fruits [17].

Most lignin activators characterized to date are from the MYB family. Among these, *MYB46* and *MYB83* have been identified as the direct targets of *SND1* and its close homologues; they function as master regulators in secondary wall formation [38] and have orthologues in several plant species, including poplar (*Populus trichocarpa*) [39] and *apple* (*Malus x domestica Borkh.*) [40]. *MYB58* and *MYB63* are transcriptional activators of lignin biosynthetic genes. Zhou et al. [41] found that *MYB58* and *MYB63* are specifically expressed in cells undergoing lignification; overexpression of *MYB58* and *MYB63* could induce the ectopic deposition of lignin, whereas dominant repression could reduce secondary wall thickening and lignin deposition. In addition, *MYB20*, *MYB42*, *MYB43*, and *MYB85* are transcriptional regulators that directly activate lignin biosynthesis genes [42]. Li et al. showed that *PtrMYB152* is involved in the positive regulation of lignin biosynthesis in poplar [43]; however, *MYB31* in banana (*Musa nana Lour.*) acts as a negative regulator of lignin biosynthesis [44]. Xu et al. [16] showed that *EjMYB1* and *EjMYB2* recognize and bind to the AC element of the *Ej4CL* promoter, thereby regulating lignin biosynthesis in loquat. *EjMYB8* has also been found to regulate lignin biosynthesis in loquat fruits [16]. In orange, *CsMYB330* is identified as a transcriptional activator, whereas *CsMYB308* appears to be a transcriptional repressor, and they play important roles in the lignification of citrus fruit juice sacs [20]. Additionally, *CsMYB85* binds the *CsMYB330* promoter and regulates its expression level, and when *CsMYB85* is overexpressed in fruit juice sacs, the *Cs4CL1* expression level and lignin content are significantly increased [45]. In pomelo, Shi et al. found that overexpressed *CgMYB58* led to significant lignin accumulation and upregulated the expression of 19 lignin biosynthetic genes [46].

Weighted gene co-expression network analysis (WGCNA) was used to analyse the correlation between physiological indicators and DEGs (differentially expressed genes) [47]. This method has been applied to the study of different plants, such as the immune regulatory network of the salicylic acid stress response in plants [48], and gene networks underlying cannabinoid and terpenoid accumulation in cannabis [49], and the regulatory network of defence hormone signalling [50]. Previously, we have demonstrated that lignin metabolism plays an important role during juice sac granulation, as lignin contents were also increased [14]. In addition, key genes involved in lignin metabolism are expressed specifically in granulated juice sacs [21]. In this study, to investigate the mechanisms of juice sac lignification in ‘SH’, we used fruit juice sacs near the core (NC) and far away from the core (FC) at three stages (S1, S4 and S8) as research materials. RNA sequencing (RNA-seq) was used to screen genes and TFs associated with lignin biosynthesis to explore their expression levels, biosynthesis, metabolic mechanism and regulation during lignin metabolism in pomelo. These results provide an important theoretical basis for studying the mechanism of fruit juice sac granulation.

## 2. Materials and Methods

### 2.1. Plant Materials

**Figure 1:**
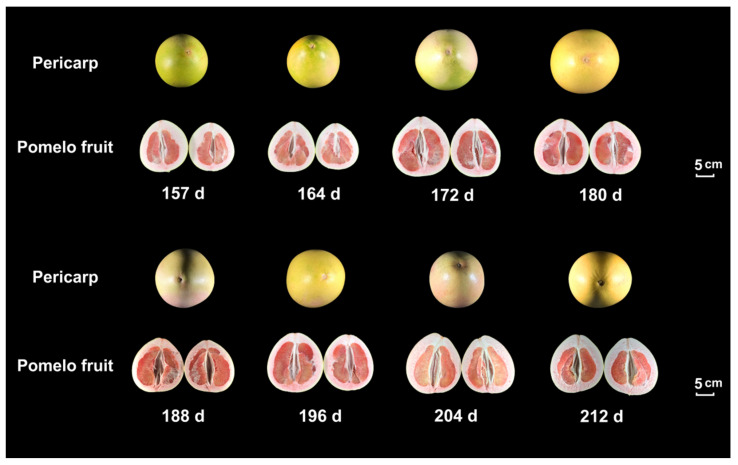
The exterior and interior characteristics of pomelo fruits at different developmental stages. Fruit samples were picked at 157, 164, 172, 180, 188, 196, 204, and 212 days post anthesis (DPA).

‘Sanhongmiyou’ was harvested from plants grown in the pomelo-producing areas in Fuqing City, Fujian Province, China. Fruit samples were picked at 157, 164, 172, 180, 188, 196, 204, and 212 days post-anthesis (DPA) (Figure 1). Fruit without visible disease and mechanical wounding were selected for the study, and three replicates were set for all sampling points. The juice sacs from each sample were immediately separated into NC (near the core) and FC (far away from the core) juice sacs, frozen in liquid nitrogen and then stored at −80 °C.

### 2.2. Determination of Lignin Content

According to the method of Chen [51], each sample was dried to a constant weight and ground into a fine powder, and 50 mg were weighed accurately for the preparation of the extracts. Then, 5 mL of deionized water, 2 mL of 95% ethanol, 2 mL of acetone, and 2 mL of ether were used to wash the pellet 3 times, respectively. After being centrifuged for 5 min (8000× *g*) each time, the pellet was dried to a constant weight. Then, 1 mL of 25% solution of acetyl bromide was added to dissolve the precipitate, which was covered and sealed in a water bath at 70 °C for 30 min. Subsequently, 1 mL of NaOH (2 mol/L) and 0.1 mL hydroxylamine hydrochloride (7.5 mol/L) were added, and the total volume was made up to 10 mL with glacial acetic acid. After each sample was diluted 6 times, they were measured to 280 nm absorption values. All the data were obtained from three biological replicates. According to the absorbance value of each concentration gradient, the standard curve was drawn by Excel software, and the regression equation obtained was Y = 5.685X − 0.0073, where the Y-axis is the absorbance, and the X-axis is the lignin concentration.

### 2.3. RNA Extraction, cDNA Library Preparation, and Sequencing

The total RNA was extracted from each sample using a Trizol reagent kit (Invitrogen, Carlsbad, CA, USA) following the manufacturer’s instructions. Three replicates for each sample were used. The quantity, quality, and integrity of the RNA were assessed on an Agilent 2100 Bioanalyzer (Agilent Technologies, Palo Alto, CA, USA) and checked by RNase free agarose gel electrophoresis. After the total RNA was extracted, using random hexamers as a six-base random primer to synthesise the first cDNA chain, then a buffer solution was added, dNTPs, RNase H, and DNA polymerase I to synthesise the second cDNA chain. The purified double-stranded cDNAs were end repaired, poly(A) added, construction of a cDNA library and Illumina sequencing, which was completed by Gene Denovo Biotechnology Co. (Guangzhou, China). The transcriptome data has been uploaded to the NCBI Sequence Read Archive (https://www.ncbi.nlm.nih.gov/sra/ (accessed on 26 March 2022)) under the accession number PRJNA817805.

### 2.4. Reads Assembly and Functional Annotation

The raw reads were filtered by fastp [52] to remove the reads containing adapters, more than 10% of unknown nucleotides (N), and more than 50% of low quality (Q-value ≤ 20) bases. Following this, the high-quality reads from all the samples were combined and assembled using Trinity to construct unique consensus sequences. These clean reads were mapped to the reference genome using HISAT2.2.4 [53]. For each gene, the expression level was measured by fragments per kilobase of transcript per million mapped reads (FPKM). Unigenes differentially expressed between two samples were screened using a false discovery rate (FDR) of <0.05 and |log2 (fold changes)| of ≥1 as the criteria. The DEGs identified were used for Gene Ontology (GO) and Kyoto Encyclopedia of Genes and Genomes (KEGG) enrichment analysis. For gene ontology and KEGG pathway enrichment, R package ClusterProfiler version 3.18.0 (Guangzhou, China) [54] was implemented, and enrichment results were filtered with the parameters of adjusted *p*-value < 0.05.

### 2.5. Identification Genes Related to Lignin Biosynthesis Pathway

The Citrus genome project data were downloaded from the website (http://citrus.hzau.edu.cn/orange/download/index.php, accessed on 30 May 2020). The Hidden Markov Model (HMM) profile of the gene domain was downloaded from Pfam (https://pfam.xfam.org, accessed on 15 June 2021). Then we used HMMER 3.0 software with an e-value of 1 × 10^−5^ as the threshold of acquiring protein sequences from the citrus genome. The protein sequences obtained from the above described were confirmed by the Conserved Domain Database (https://www.ncbi.nlm.nih.gov/Structure/bwrpsb/bwrpsb.cgi, accessed on 20 June 2021) to ensure the presence of the gene domain.

### 2.6. Weighted Gene Co-Expression Network Analysis Factor

A gene co-expression network was built using the WGCNA package (Version 4.0.2) [47], for which the soft threshold was set as 14, and a correlation threshold value of 0.75 was applied in the modules’ merging. Genes with module membership over 0.8 were selected as the high module membership genes for each module and processed for the enrichment and co-expression study. Furthermore, genes with a high co-expression connection within the module were filtered, and a co-expression network illustration was conducted with Cytoscape version 3.7.1 [55].

### 2.7. Validation of DEGs by Quantitative Real-Time PCR (qRT-PCR)

To validate the reliability of our RNA-Seq database, 13 putative candidate functional genes and 19 TFs selected from the DEGs were further analyzed using qRT-PCR. The total RNA of 18 fruit samples was extracted using an RNA prep pure Kit (BioFlux Biotech, Hangzhou, China). First-strand cDNA was synthesized using the PrimeScript RT Reagent Kit (TaKaRa, Dalian, China). The cDNAs were synthesized from 1 µg of total RNA and diluted 10-fold for gene expression experiments. qRT-PCR was performed on a Jena qTOWER 2.2 real-time quantitative fluorescent PCR instrument with an SYBR^®^ Premix ExTaqTM (Tli RNaseH Plus) (Takara, Dalian, China). The normalized expression level of the gene in NC juice sacs at 157 DPA was used as a control value (expression set to 1), and the *β*-tublin gene was used as a reference gene for gene expression normalization [21]. All reactions were performed in independent biological and technical triplicates; relative expression analysis was performed using the 2^−∆∆CT^ method, and the specific primers were designed for these genes (Supplementary Table S1).

## 3. Results

### 3.1. Physiological Indicators of Juice Sacs in ‘SH’ during Fruit Ripening

**Figure 2:**
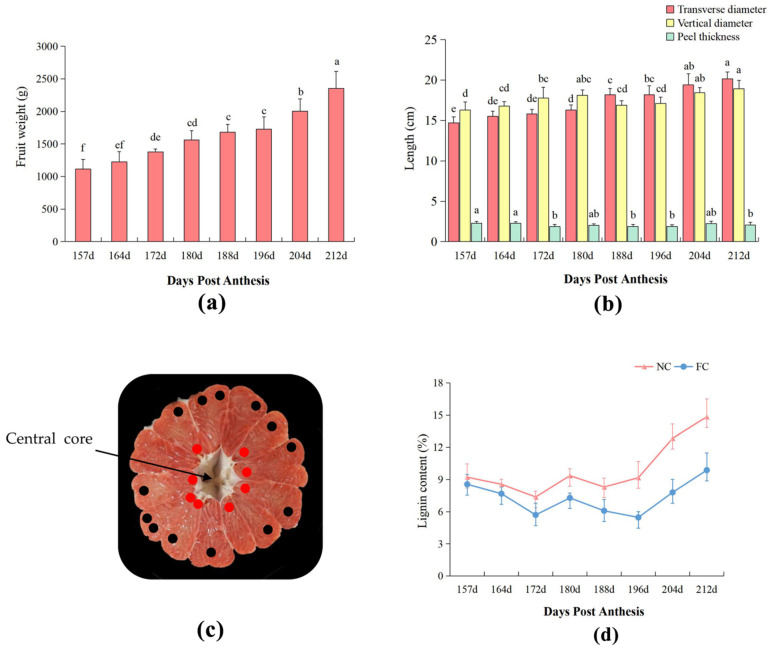
The dynamic changes in physiological indicators of ‘SH’ at different developmental stages. (**a**) Changes in fruit weight from 157–212 d; (**b**) Changes in the transverse diameter, vertical diameter, and peel thickness at different developmental stages; (**c**) Morphological diagram of a fruit cross-section, where the red dots indicate the juice sacs near the core (NC), and the black dots indicate the juice sacs far away from the core (FC); (**d**) The lignin content of fruit juice sacs in NC and FC at different development stages. Each data point is the average of three biological repeats, and different letters indicate significant differences at *p* < 0.05 as determined by a one-way analysis of variance (ANOVA) with Duncan’s multiple range test and Kruskal–Wallis test.

The exterior and interior characteristics of pomelo fruits in different developmental stages, from 157 DPA to 212 DPA, are shown (Figure 1). The weight of the ‘SH’ fruit increased during fruit ripening with an overall increasing trend. The heaviest fruit reached 2353.70 g at 212 DPA (Figure 2a). The transverse and vertical diameters also showed an increasing trend, with maximum values of 20.14 cm and 18.91 cm at 212 DPA and the minimum values of 14.70 cm and 16.28 cm on 157 DPA. The peel thickness of the fruits fluctuated between 1.82 and 2.23 cm and was significantly different (Figure 2b).

With the growth and development of the ‘SH’ fruit, the lignin content of the juice sac tissues near the core (NC) and far away from the core (FC) increased from 157 DPA to 212 DPA. The inflexion point occurred at 180 DPA, and the lignin content of NC and FC juice sacs first decreased and then increased, suggesting that the sacs were in the early stage of expansion and not fully mature. The lignin content in NC and FC juice sacs reached a maximum at 212 DPA; additionally, the lignin content of the juice sacs in NC (14.8%) was much higher than that in FC (9.9%) at 212 DPA (Figure 2d).

### 3.2. Quality Analysis of the RNA-seq Results

Eighteen cDNA libraries were constructed from the total RNA of NC and FC juice sacs at 157 DPA (stage 1, S1), 180 DPA (stage 4, S4), and 212 DPA (stage 8, S8). The raw sequencing data were filtered and assembled. The quality assessment of transcriptome sequencing showed that the proportion of clean data was higher than 99% in all 18 samples, indicating that gene expression profiles were of high quality. A total of 35.55 million to 46.30 million original sequences were sequenced from 18 samples of NC and FC juice sacs at three developmental stages. After quality control, 35.43 million to 46.18 million valid sequences were obtained, accounting for 99.49–99.88% of the raw sequences. A total of 32.22 million to 43.59 million sequences were successfully aligned with the sweet orange genome. Among the reads that uniquely mapped to the reference genome accounted for 92.87–94.22% (Supplementary Table S2).

### 3.3. Identification and Functional Annotation of DEGs during Fruit Development

**Figure 3:**
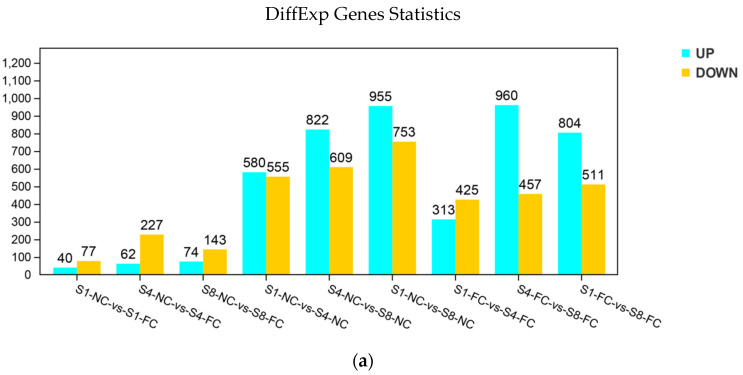

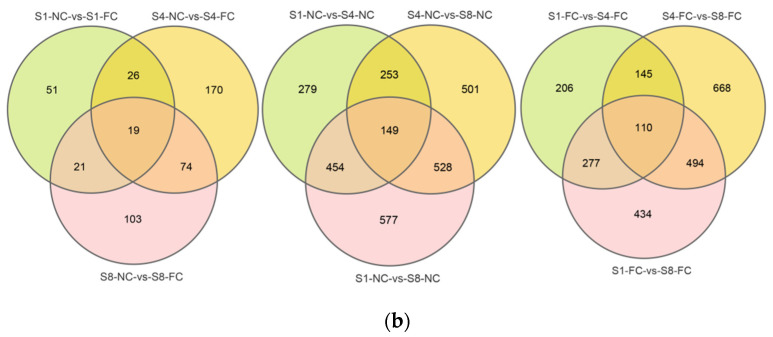
Statistical map of differential expression gene (DEGs). (**a**) Numbers of DEGs in pairwise comparisons. The X-axis indicates the name of each group; the Y-axis shows the gene number of genes within each group. NC indicates the fruit juice sacs near the core (NC), and FC indicates the fruit juice sacs far away from the core (FC) at three stages (S1, S4 and S8); (**b**) Venn diagram of shared DEGs between each group.

To identify variations in gene expression between the NC and FC juice sacs at different stages (S1, S4 and S8), we identified 8367 differentially expressed genes (DEGs) (|logFC > 1|, FDR < 0.01) based on the pairwise comparisons among the nine groups. In the normal stage (S1), there were 117 DEGs in NC and FC juice sacs. In the gelation stage (S4), there were 289 DEGs in NC and FC juice sacs. In the lignification stage (S8), there were 217 DEGs in NC and FC juice sacs. These results indicated that most genes are expressed in the gelation stage. There were 1135 DEGs (580 upregulated and 555 downregulated in the S1 vs. S4 comparison in NC juice sacs), while in FC juice sacs, there were 738 DEGs (313 upregulated and 425 downregulated). There were 1708 DEGs (955 upregulated and 753 downregulated) in the S1 vs. S8 comparison in NC juice sacs, while in FC juice sacs, there were 1315 DEGs (804 upregulated and 511 downregulated). These genes may play an important role in fruit granulation, especially in the late maturity stage (Figure 3a).

Moreover, the results for the DEGs visualized via a Venn diagram revealed that 19, 149, and 110 genes were specifically expressed in the three groups, respectively. Among these DEGs, pairwise comparisons of NC and FC juice sacs in three stages revealed 19 shared DEGs, indicating tissue specificity in the fruit. Pairwise comparisons of different stages of NC juice sacs revealed 149 shared DEGs, indicating that NC juice sacs gradually showed specificity as the stages progressed. Pair comparison of different stages of FC juice sacs revealed 110 shared DEGs, indicating that FC juice sacs gradually showed specificity as the stages progressed (Figure 3b).

**Figure 4:**
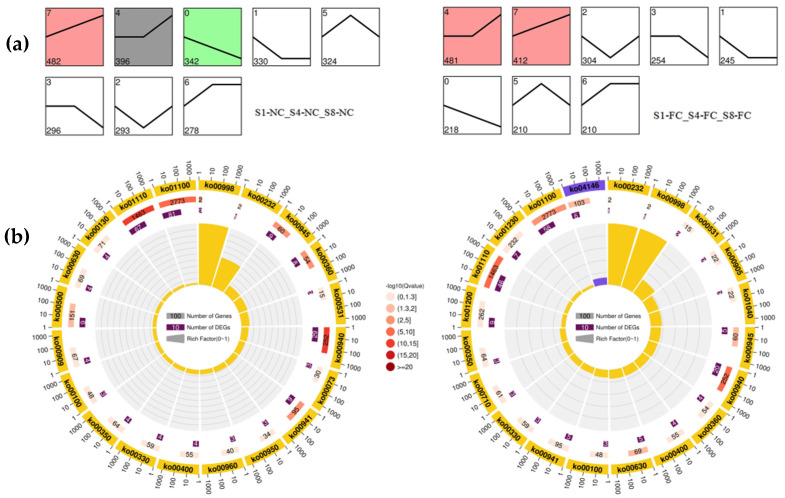
(**a**) Trend analysis of DEGs divided into eight clusters based on expression patterns. Profiles are ordered based on the number of genes assigned; (**b**) KO enrichment circle diagram. Outermost circle: the top 20 enriched pathways with the number of genes shown outside the circle. Different colours represent different classes. Second circle: the number of pathways and the Q values in the background gene. Longer bars represent a higher number of genes, while the smaller the Q value, the redder the colour; Third circle: the number of differentially expressed genes in the pathway. Fourth circle: the RichFactor value of each pathway (the number of differentially expressed genes in the pathway divided by the total number of genes), where each grid represents 0.1.

STME [56] was used for trend analysis to enrich the GO and KEGG functions of the unigenes in the two groups. GO analysis showed that DEGs in NC and FC juice sacs at developmental stages were mainly concentrated in molecular functions, cell composition, and biological processes (Figure S2). DEGs were mainly concentrated in ‘cell wall macromolecule metabolic process’ (GO:0044036), ‘cell wall polysaccharide metabolic process’ (GO:0010383), ‘xylan metabolic process’ (GO:0045491), ‘hemicellulose metabolic process’ (GO:0010410), and ‘cell wall organization or biogenesis’ (GO:0071554) (Supplementary Table S3). According to the expression trend analysis of the NC and FC juice sacs at the three stages, we found that Profile 7 was extremely significant (Figure 4a). In NC juice sacs, Profile 7 contained 482 DEGs, of which 81 DEGs were enriched in ‘metabolic pathways’ (ko01100), 67 DEGs were enriched in ‘biosynthesis of secondary metabolites’ (ko01110), and 29 DEGs were enriched in ‘phenylpropane biosynthesis’ (ko00940). In FC juice sacs, Profile 7 contained 412 DEGs, of which 58 DEGs were enriched in ‘metabolic pathways’ (ko01100), 46 DEGs were enriched in ‘biosynthesis of secondary metabolites’ (ko01110), and 20 DEGs were enriched in ‘phenylpropane biosynthesis’ (ko00940) (Figure 4b).

### 3.4. WGCNA Shows the Enrichment of Lignin Metabolism Pathways during NC and FC Juice Sac Development in Pomelo

#### 3.4.1. Screening of DEGs Highly Correlated with Lignin Content

**Figure 5:**
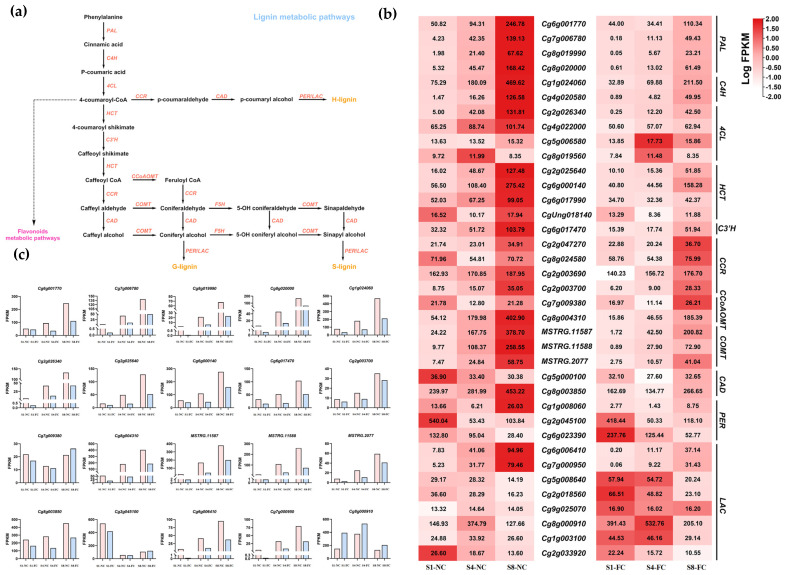
(**a**) The lignin metabolic pathway in pomelo; (**b**) Heatmap of lignin biosynthesis genes in NC and FC juice sacs at three stages; (**c**) The expression level of 20 DEGs related to lignin metabolism was based on the FPKM value. FPKM values were the means of three replications.

**Figure 6:**
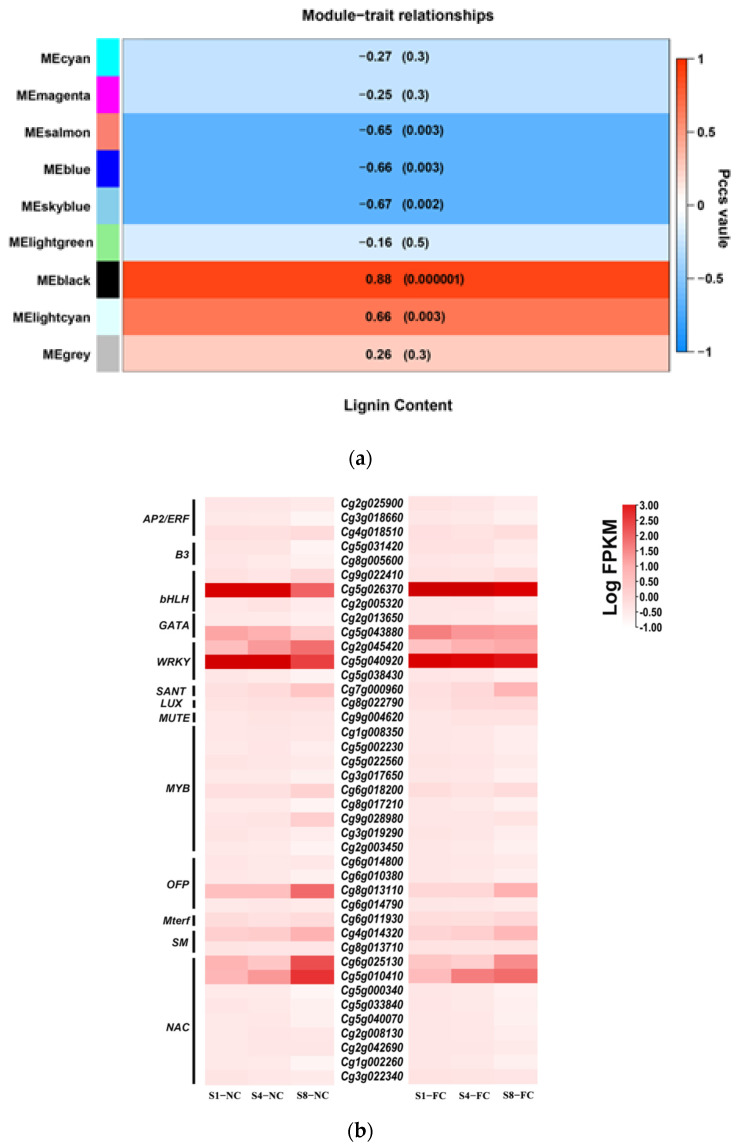
Weighted Gene Co-Expression Network Analysis (WGCNA) analysis of DEGs. (**a**) The module-sample correlations between DEGs and lignin content. Corresponding *p* values of module-sample correlations are indicated in parentheses. The panel on the left side shows the nine modules: cyan module (MEcyan), magenta module (MEmagenta), salmon module (MEsalmon), blue module (MEblue), skyblue module (MEskyblue), lightgreen module (MElightgreen), black module (MEblack), lightcyan module (MElightcyan), grey module (MEgrey). The colour scale on the right side shows module-trait correlations from −1 (blue) to 1 (red). (**b**) Heatmap of TFs in NC and FC juice sacs at three stages.

To analyse the changes in lignin content and the co-expression of DEGs, RNA-seq was performed to analyse the DEGs in NC and FC juice sacs at three stages (S1, S4, and S8). A total of 37 lignin biosynthesis genes were obtained by hidden Markov model (HMM) analysis, including *CmPALs*, *CmC4Hs*, *Cm4CLs*, *CmHCTs*, *CmC3Hs*, *CmCCRs*, *CmCCoAOMTs, CmCOMTs*, *CmCADs*, *CmPERs*, and *CmLACs*, and 20 of genes were significant DEGs (Figure 5). Overall, we found that the expression levels of each subtype of DEG involved in lignin biosynthesis showed the same expression trends, and the juice sacs of NC and FC showed an overall upward trend at the three stages, where the expression level of NC juice sacs was much higher than that of FC juice sacs. At the same time, WGCNA was used to analyse the association between the co-expressed gene modules formed by all DEGs and the lignin content. The modules with similar expression patterns were then merged according to the similarity of the module feature values, and nine modules were identified: MEcyan, MEmagenta, MEsalmon, MEblue, MEskyblue, MElightgreen, MEblack, MElightcyan, and MEgrey modules. Among these modules, we found that the ME display of the black module was highly positively correlated with the lignin content under juice sac granulation, and the correlation coefficient was 0.88. The black module contains 1287 genes that might play an important role in lignin synthesis (Figure 6a).

To systematically study the regulatory networks of lignin metabolism-related genes and transcription factors, we used Pearson correlation analysis to screen out highly correlated TFs using 11 DEGs related to lignin synthesis in the black module. We plotted all pairs of adjustment relationships using a Pearson correlation coefficient threshold greater than 0.7. These TFs belong to 13 transcription factor families, and the main transcription factor families were MYB, NAC, OFP, ERF, and bHLH. Heatmap analysis among three biological repeats showed that the expression patterns of transcription factors in NC and FC juice sacs were different during the three stages (Figure 6b).

#### 3.4.2. Differential Gene Expression Validation by qRT-PCR Analysis

**Figure 7:**
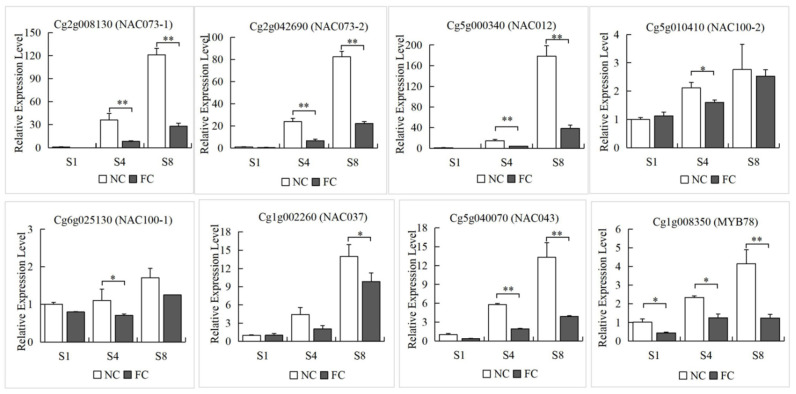

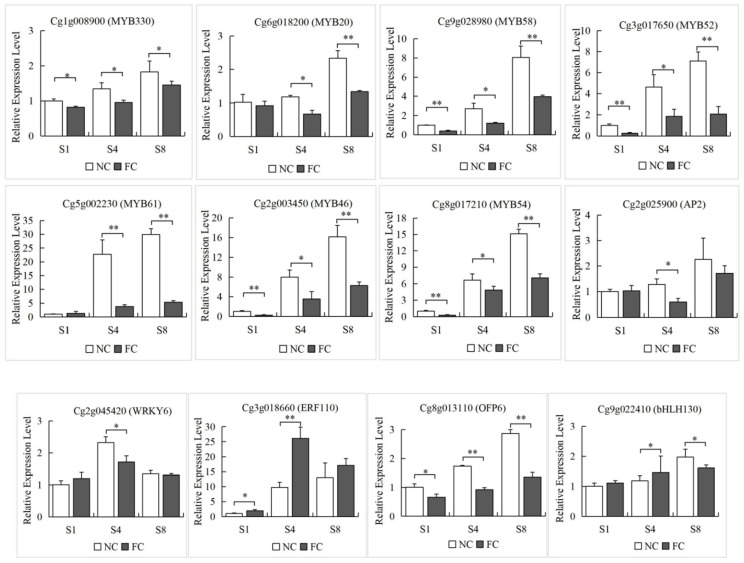
Validation of expression patterns by qRT-PCR of selected DEGs identified in the RNA-seq analysis. The Y-axis represents the relative expression levels. The corresponding bar plot shows the standard deviations (denoted as error bars). The significance of differences compared with FC juice sacs is indicated. Student’s *t*-test: *: 0.01 < *p* < 0.05; **: 0.001 < *p* < 0.01.

**Figure 8:**
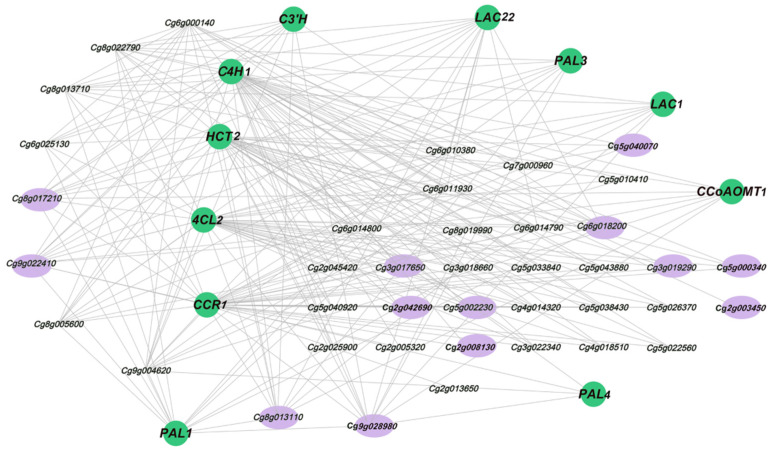
Construction of regulatory networks of lignin metabolism genes and transcription factors (TFs). Green nodes indicate structural genes related to lignin biosynthesis, and purple nodes indicate candidate TFs that may regulate the lignin metabolism.

With the development of the fruit, the granulation phenomenon gradually became serious, and most of them were found in the NC juice sacs. The 20 DEGs were screened according to the GO analysis and the expression patterns of genes in RNA-seq, and qRT-PCR was performed to verify the DEGs in NC and FC juice sacs at three stages. We found that the expression level of DEGs showed an overall upward trend from S1 to S8. It is worth noting that the expression level of the genes in NC juice sacs was significantly higher than that in FC juice sacs, which was consistent with the trend for lignin accumulation. The gene expression levels of *MYB46*, *MYB330*, *MYB61*, *MYB20*, *MYB58*, *MYB78*, *MYB52*, *MYB54*, *NAC012*, *NAC073-1*, *NAC73-2*, *NAC043*, *OFP6*, and *bHLH130* showed a more significant difference in NC and FC juice sacs at the S8 stage. In contrast, there were no significant differences in *AP2*, *WRKY6*, *ERF110*, *NAC100-1*, and *NAC100-2* (Figure 7). Then we constructed a lignin metabolism regulatory network based on the screened 11 lignin synthesis-related genes and 13 transcription factors from the black module (Figure 8).

### 3.5. Verification of the Selected DEGs through qRT-PCR

To further verify the reliability of the transcriptome data, 13 enzyme-encoding genes (*CCR*, *HCT*, *4CL*, *C4H*, *LAC*, *PAL*, *C3H*, *COMT*, and *CCoAOMT*) associated with lignin biosynthesis were analysed by qRT-PCR. The relationship between the RNA-Seq and qRT-PCR in NC and FC juice sacs at three stages was analysed, respectively, based on Pearson’s correlation analysis. Results showed that the relative expression trend for these genes was highly consistent with the RNA-seq data, and correlation values were higher than 0.9, which further demonstrated the reliability of the transcriptome data (Supplementary Figure S3). These results confirmed that our transcriptome data could serve as a foundation for further analysis.

## 4. Discussion

Previous studies have shown that lignin accumulation is significantly positively correlated with the expression levels of the key genes in phenylpropane metabolic pathways, lignin-specific synthetic pathways, and downstream pathways of lignin synthesis [57]. She [14] demonstrated that lignin metabolism plays an important role in juice sac granulation, as lignin contents increased during fruit development. In addition, key genes involved in lignin metabolism are expressed specifically in granulated juice sacs [21]. Juice sac granulation in pomelo often occurs at the late stage of fruit ripening, which is characterized by abnormally enlarged, stiffened, dried, and lignified juice sacs, and the most severe granulation often occurs in tissues near the central core of the fruit [58]. This granulation phenomenon was most common in ‘SH’ and ‘HR’ mutant pomelo. In this study, we used fruit juice sacs near the core (NC) and far away from the core (FC) at various stages as research materials. We found that the lignin content in NC and FC juice sacs increased from 157 DPA to 212 DPA and reached a maximum of 212 DPA. Additionally, the lignin content of NC juice sacs was higher than that of FC juice sacs. This study showed that the lignin components gradually accumulated during the development of pomelo fruits, especially in the NC juice sacs (Figure 2d).

Comparative transcriptome analysis is a useful approach for discovering important clues about gene functions and the molecular basis of developmental processes [59]. In this study, we used 18 cDNA libraries which were constructed from the total RNA of NC and FC juice sacs at 157 DPA (stage 1, S1), 180 DPA (stage 4, S4), and 212 DPA (stage 8, S8). After analysing the DEGs, we found that there were 8367 genes with significant differences; these genes were mainly concentrated in the pairwise comparison of S1-NC vs. S8-NC, S4-NC vs. S8-NC and S4-FC vs. S8-FC, indicating that these DEGs may play an important role in fruit juice sac granulation, especially in the late stage of development (Figure 3a). The results of the GO and KEGG pathway enrichment analyses revealed that the DEGs were mainly concentrated in ‘cell wall macromolecule metabolic process’, ‘cell wall polysaccharide metabolic process’, and ‘cell wall organisation or biogenesis’. According to the expression trends within the NC and FC juice sacs at the three stages, metabolic pathway enrichment analysis of these differential metabolites showed significant enrichment of ‘phenylpropane biosynthesis’ and ‘biosynthesis of secondary metabolites’ in all comparison groups (Figure 4b). In plants, lignin is one of the most important components of the cell wall, and the expression and transcriptional regulation of lignin biosynthesis-related genes have been studied widely in *Arabidopsis* and other plants [24,60,61].

Lignin is synthesized through the phenylpropane pathway, and previous studies have revealed that key regulatory genes, such as *PAL*, *C4H*, *4CL*, *CAD*, and *POD*, are important genes of the lignin synthesis pathway [62,63]. Four 4CL proteins have been identified in *Arabidopsis* and may have distinct functions in the phenylpropanoid metabolism pathway. In addition, *4CL1* is the major *4CL* and the 4cl1 mutant shows a decreased lignin content [64]. In loquat, *PAL*, *4CL*, and *CAD* showed a correlation with fruit lignification [65,66], and *CAD1* was first identified as a key candidate in the regulation of chilling injury-related lignification [66]. The expression of other genes, such as *CCoAOMT*, also showed a positive correlation with lignin content [67]. Lignin accumulation is positively associated with the degree of fruit juice sac granulation. Pan et al. [68] demonstrated that elevated PAL and CAD activities were accompanied by an increase in the degree of granulation of pomelo fruits. In addition, Wu et al. [1] found that lignin in granulated juice sacs was characterized by an extremely high abundance of G units. Some lignin synthesis pathways, such as *CmPAL*, *CmC4H*, *CmHCT*, *CmC3H*, *CmCCoAOMT1*, *CmCCR*, and *CmCAD1*, were also specifically expressed in granulated juice sacs [21]. A total of 37 lignin biosynthesis genes were obtained by HMM analysis, and 20 genes were significant DEGs based on the RNA-seq database (Figure 5).

WGCNA was used to analyse the association between the co-expressed gene modules formed by all DEGs and the lignin content. The black module was highly positively correlated (0.88) with the lignin content and contained 1287 DEGs, indicating that these genes might play an important role in lignin synthesis (Figure 6a). In the black module, a total of 11 DEGs, *PAL1 (Cg6g001770)*, *PAL3 (Cg8g019990)*, *PAL4 (Cg8g020000)*, *C4H1 (Cg1g024060)*, *4CL2 (Cg2g026340)*, *HCT2 (Cg2g025640)*, *C3H (Cg6g017470)*, *CCR1 (Cg2g003700)*, *CCoAOMT1 (Cg8g004310)*, *LAC1 (Cg6g006410)*, and *LAC22 (Cg7g000950)*, which are related to the lignin biosynthesis were identified as hub genes. By combining the Pearson correlation analysis (Figure 6b) and the gene expression level results (Figure 7), we selected several candidate transcription factors: *MYB78 (Cg1g008350)*, *MYB330 (Cg1g008900)*, *MYB46 (Cg2g003450)*, *MYB52 (Cg3g017650)*, *MYB61 (Cg5g002230)*, *MYB20 (Cg6g018200)*, *MYB58 (Cg9g028980)*, *bHLH130 (Cg9g022410)*, *NAC012 (Cg5g000340)*, *NAC043 (Cg5g040070)*, *NAC073-1 (Cg2g008130)*, *NAC073-2 (Cg2g042690)*, and *OFP6 (Cg8g013110)*. Among these TFs, the MYB and NAC families contained the greatest number of TFs related to DEGs, indicating that these TFs might be key regulators of the formation of lignin. Previously, several MYB and NAC TFs have been reported to play an important role in the regulation of lignin synthesis as both transcriptional activators and repressors [32,69]. Zhong et al. [70] demonstrated that *SND1* and its homologous genes could activate their direct targets through binding to an imperfect palindromic 19 bp consensus sequence, called the secondary wall NAC-binding element (SNBE), to regulate the lignin biosynthesis. *EjNAC1* and *EjNAC3* belong to the NAC family; *EjNAC3* regulates *EjCAD*-like expression and influences lignin content [37]. Xu et al. found that *EjNAC1*, a homologue of *AtVND6* and *AtVND7*, was also shown to act as a positive upstream regulator of loquat fruit lignification [36]. Overexpression of *BpNAC012* activated the expression of SCW- associated genes, resulting in ectopic deposition in the stem epidermis [71]. Additional reports implicated several MYB TFs as secondary master regulators of SCW formation [38]. The reports revealed that only specific MYB TFs with R2R3 domains could bind to one or more AC elements (AC-I, AC-II and AC-III) and regulate the expression of lignin synthesis genes [72]. *AtMYB58* and *AtMYB63* are downstream target genes of NAC TFs and are closely related to lignin synthesis. The inhibition of *AtMYB58* and *AtMYB63* expression by RNAi resulted in a significantly decreased lignin content and reduced secondary wall thickening [41]. In addition, MYB20, MYB42, MYB43, and MYB85 are transcriptional regulators that directly activate lignin biosynthesis genes [42]. *EjODO1* has high sequence homology with *AtMYB20* and can transactivate the promoters of *EjPAL1*, *Ej4CL1*, and *Ej4CL5*, thus regulating lignin biosynthesis in developing loquat fruit [73]. Many transcription factors, such as *CgMYB58*, *CsMYB330*, *CsMYB308*, and *CsMYB85*, have been investigated in relation to the regulation of fruit juice sacs granulation [20,45,46].

Based on this study, we identified *MYB78*, *MYB330*, *MYB46*, *MYB52*, *MYB61*, *MYB20*, *MYB58*, *bHLH130*, *NAC012*, *NAC043*, *NAC073-1*, *NAC073-2*, and *OFP6* as candidate TFs involved in lignin synthesis. In this study, we present new evidence that NAC and MYB TFs play important roles in the lignification of citrus fruit juice sacs; these observations indicate that candidate genes have transcriptional regulatory functions similar to those of their homologous genes in other plants, such as Arabidopsis and loquat. Accordingly, we believe that the research present herein will make an important contribution to understanding the regulatory mechanisms of lignin metabolism in citrus fruits.

## 5. Conclusions

In our study, the physiological indicators and transcriptome sequences of NC and FC juice sacs of ‘SH’ were studied at different developmental stages. By transcriptome data analysis, we identified 20 DEGs that are involved with the lignin synthesis pathway. In addition, 13 differently expressed transcription factors were screened by WGCNA and qRT-PCR analysis. Consequently, these genes named MYB, NAC, OFP6, and bHLH130 could be used as candidate genes for future study. Our research will supply a valuable gene resource for guided plant breeding.

## Data Availability

The 18 samples from the RNA-seq data were submitted to the NCBI Sequence Read Archive (https://www.ncbi.nlm.nih.gov/sra/, accessed on 26 March 2022) under the accession number PRJNA817805.

## Supplementary Materials

The following supporting information can be downloaded at: https://www.mdpi.com/article/10.3390/genes13050845/s1. Supplementary Table S1: List of Primers used for gene qRT-PCR. Supplementary Table S2: High quality clean reads mapped to the reference sequence. Supplementary Table S3: Results of GO enrichment analysis for the differentially expressed genes. Supplementary Table S4: Candidate DEGs relate to the lignin synthesis. Supplementary Figure S1: Difference comparison Volcano chart. Supplementary Figure S2: Gene Ontology (GO) assignment of DEGs. Supplementary Figure S3: Validation of expression patterns by qRT-PCR of selected DEGs identified in the RNA-seq analysis.
