# Supplementary material for: Transcriptome Analysis Reveals Candidate Lignin-Related Genes and Transcription Factors during Fruit Development in Pomelo (Citrus maxima)
Source: Genes (Basel). 2022 May 9;13(5):845. doi: 10.3390/genes13050845 (PMC9140673; doi:10.3390/genes13050845)
Supplement: Supplementary file 1 [file genes-13-00845-s001.zip › genes-1678026-supplementary.pdf]

Supplementary Table S1. Primers used for DEGs qRT-PCR

**Primer Name**	***Forward primer (5′ to 3′)***	***Reverse primer (5′ to 3′)***	**Primer Name**	***Forward primer (5′ to 3′)***	***Reverse primer (5′ to 3′)***
AP2-F/R	TAGAGGCGTAGCAAGACACC	ACTCTATTGCTGCCCTGTCA	PAL6-F/R	CAACACCCGTCTAGCCATTG	CAGGATTGGCGAGGAATTGG
ERF110-F/R	TTCCGGTCAAAGAGCTGCTA	GAAGCAGAAGCTGGAGAAGC	PAL3-F/R	CAGCTTCGGAGTCGTTGAAG	AACCCAATCACTGCTAGCCT
BHLH130-F/R	CAACGGAAGATGCCCAAGAG	TAGAGTTGCCTGCCACATCA	PAL4-F/R	TGTTCCGAGCTCCAGTTTCT	TCTCCTCCAAGTGCCTCAAG
WRKY6-F/R	AAGTTCAACGTTGTGCGGAA	GCAGCTGTTGTTGTTGATGC	C4H2-F/R	GGTACTACACACGCAAGGTG	CATGCTCCCACATGTCACTG
OFP6-F/R	GTACTCAGCCCTTGCCCTAA	AGAATTCAGCTGCAAGAAGCA	4CL2-F/R	CCGGCGACATTGGTTACATT	AAATGCAACGGGAACCTCAC
MYB78-F/R	GGGAAACAGGTGGTCGAAAC	TTTGAACTCGGGTCCTCCAA	HCT2-F/R	GCCGTATCGAGATTGACTGC	GTTGGGATGAGCTGCTTCAG
MYB61-F/R	CACCACTCTTGCTGCAATCA	CCGACAACTCTTACCGCATC	CCR1-F/R	ACCGGAGCATCTGGTTACAT	TGCAAGTAAGTGTCCCGTCT
MYB20-F/R	GCCATGGACAGCTGAAGAAG	CAAGCTTGGGAACAGCTCTC	LAC1-F/R	TCAACCAAGCTTTGCAGTCC	TGTGACGAGCAACTGGAGAT
MYB54-F/R	GCTGTCAAGAATCACTGGCA	ATCAGTGCAACCGGTTTGTG	LAC22-F/R	GCAGGATGGACAGCAATCAG	TCGGAAGGTCACTAGGAGGA
MYB52-F/R	ATGGTAGCTATCGCGGTTCT	TGTGATGGCGAGTTATCGGA	C3H-F/R	CACGTCAACGTATGGGCAAT	GGCCTTTCATGTCCACATCC
MYB58-F/R	CAGCCGAACATGGTTCCAAT	GGTCCACTTCTGGTTTCAGC	COMT1-F/R	TCGGATGAAGAGGCAAACCT	GGTATGGGCATCTGGGTTCT
MYB46-F/R	ATAACGGCAACGGTAACAGC	GCCCAGCATTTCTAGCAACA	CCoAOMT1-F/R	CACAAGAGCCTGCTCCAATC	TCAAGAACTGCCCTTCGTCT
MYB330-F/R	GCAGTGGTGTCACAACAGAA	AGCGCCGGTACTATGGTTAT	NAC043-F/R	CGAACTCCTCTGGTAGCCAA	GATGATGACTCCGTCTCCGT
NAC073-2-F/R	ATGAGGAGAACCCGAATGGG	GGAACCTGCAAACCTTCTGG	NAC037-F/R	TCCGACTGATGAGGAACTCG	GATCCGGCATCTCTCTTGGA
NAC073-1-F/R	TGGAGTGAGCAGTAGTGGAG	TCTCTCAATCGTTTGCACAT	MYB308-F/R	CTTCACGCACTTCTAGGCAA	CTGTGGTGCAACCGATGATT
NAC012-F/R	GAGAGTCGTCGGAGTCCATT	GTTGCTGGAACCAATCAGCA	*β*-tubIin-F/R	ACATCCCGCCTAAGGGTCTG	TTCCTCCGAAACATAGCCGTA

Supplementary Table S2. High quality clean reads mapped to the reference sequence

Sample	Raw Data	Clean Data	Effective reads ratio (%)	Unique_Mapped (%)
S1-NC-1	37275792	37223002	99.86%	35291523 (94.22%)
S1-NC-2	35551026	35433468	99.67%	33728153 (93.92%)
S1-NC-3	40145670	40089516	99.86%	37731930 (94.08%)
S1-FC-1	36706118	36517526	99.49%	32222597 (93.85%)
S1-FC-2	39090136	38962908	99.67%	36713736 (93.89%)
S1-FC-3	38455878	38329364	99.67%	36590651 (94.08%)
S4-NC-1	43537492	43418702	99.73%	40549044 (94.08%)
S4-NC-2	46297988	46180142	99.75%	43594079 (93.75%)
S4-NC-3	37479334	37315758	99.56%	35305661 (93.96%)
S4-FC-1	36520704	36475724	99.88%	34460993 (93.86%)
S4-FC-2	39305690	39209430	99.76%	36247617 (93.71%)
S4-FC-3	45112358	44885410	99.50%	41818381 (92.87%)
S8-NC-1	45782402	45614302	99.63%	42679204 (93.40%)
S8-NC-2	40826380	40662864	99.60%	38469423 (93.55%)
S8-NC-3	45333420	45158990	99.62%	42500804 (93.83%)
S8-FC-1	42728046	42568110	99.63%	40167633 (93.26%)
S8-FC-2	40395818	40251408	99.64%	38187507 (93.80%)
S8-FC-3	37439170	37303140	99.64%	34895085 (93.63%)

[1] The granulation of juice sacs at not seen stage (S1), granulation begins to occur (S4), and most severe granulation (S8). Three biological replicates of each sample; All Reads Num: total number of reads after ribosome removal; Unique Mapped Reads: number of reads on unique alignment with reference sequence; Multiple Mapped Reads: number of reads on multiple alignments with reference sequence; Mapping Ratio = (Unique Mapped Reads + Multiple Mapped Reads)/All Reads Num.

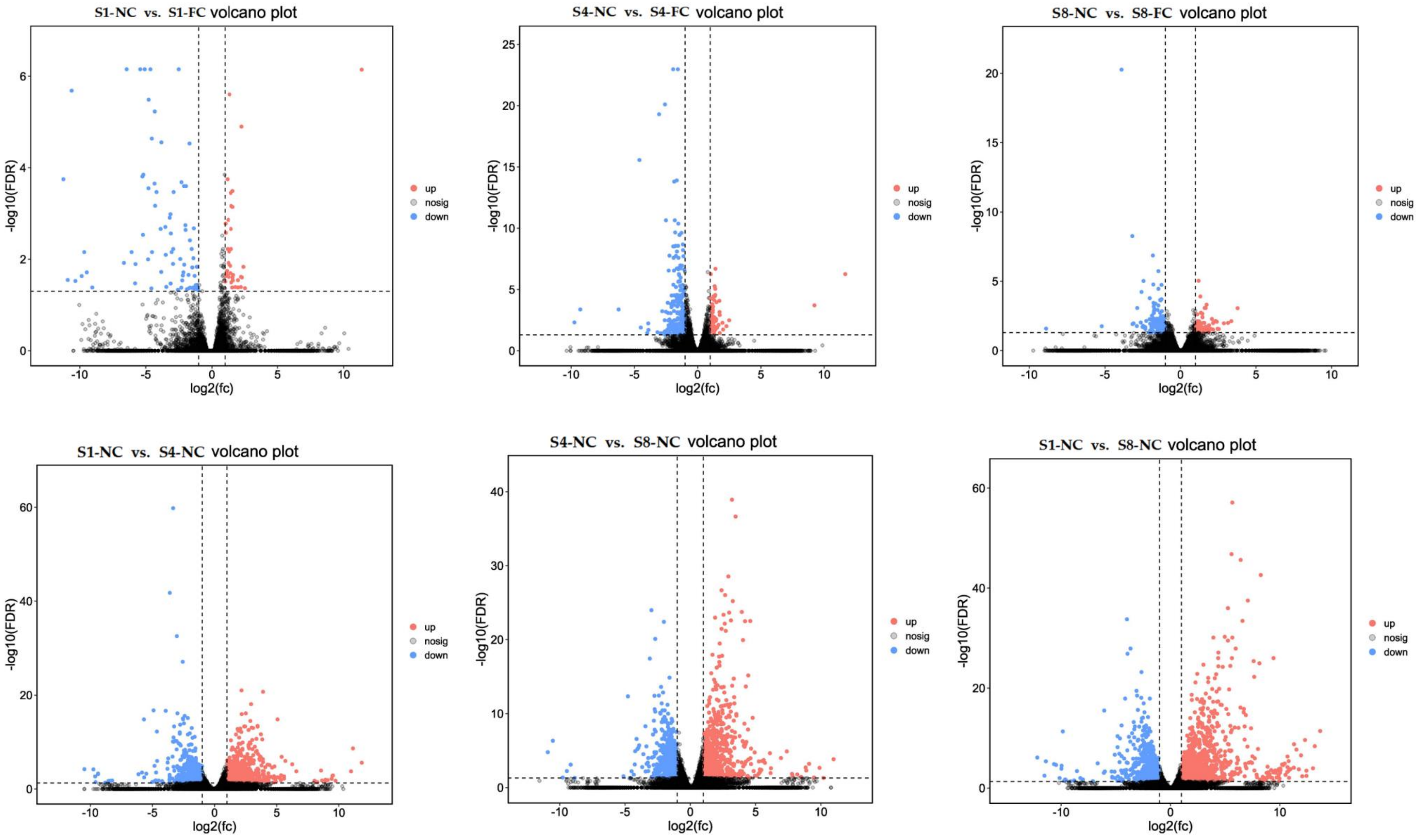
S1-NC vs. S1-FC volcano plot
S4-NC vs. S4-FC volcano plot
S8-NC vs. S8-FC volcano plot
S1-NC vs. S4-NC volcano plot
S4-NC vs. S8-NC volcano plot
S1-NC vs. S8-NC volcano plot
-log10(FDR)
log2(fc)
up
nosig
down

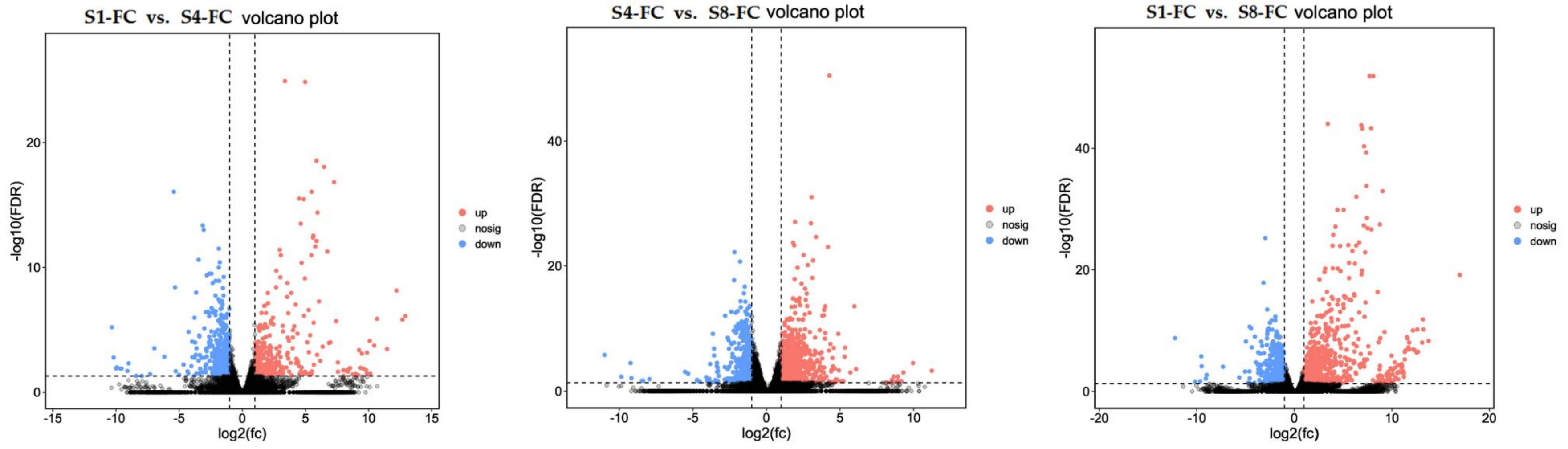

Supplementary Figure S1. Difference comparison Volcano chart. The abscissa represents the logarithm of the fold difference between the two groups, the ordinate represents the negative Log10 value of the FDR of the difference between the two groups, and the red (group_2 expression is up-regulated relative to group_1) and blue (expression is down-regulated) points represent the gene's There is a difference in expression (the criterion is FDR <0.05, and the difference is more than twice the fold), and the black point is no difference.

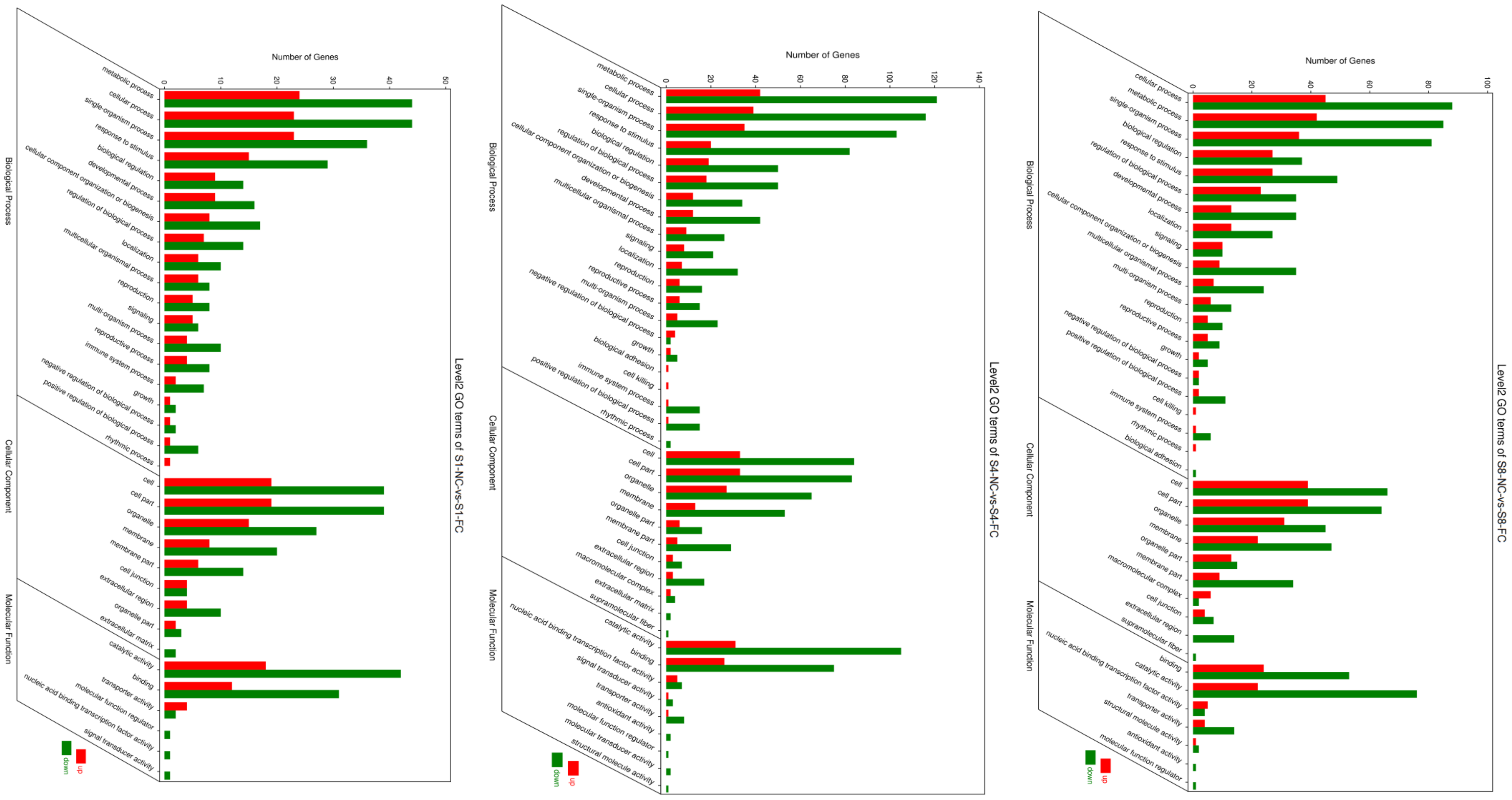

Supplementary Figure S2. Gene Ontology (GO) assignment of DEGs. Red and green bars show the GO distributions of up-regulated and down-regulated DEGs, respectively.

Supplementary Table S3. Results of GO enrichment analysis for the differentially expressed genes

Grope	GO ID	Description	Number of enriched DEGs	All DEGs	pvalue	qvalue
S1-NC-vs-S1-FC	GO:0044036	cell wall macromolecule metabolic process	20 (23.26%)	266 (1.75%)	0.000000	0.000000
	GO:0010383	cell wall polysaccharide metabolic process	19 (22.09%)	238 (1.57%)	0.000000	0.000000
	GO:0045491	xylan metabolic process	18 (20.93%)	210 (1.39%)	0.000000	0.000000
	GO:0010410	hemicellulose metabolic process	18 (20.93%)	211 (1.39%)	0.000000	0.000000
	GO:0071554	cell wall organization or biogenesis	27 (31.4%)	746 (4.92%)	0.000000	0.000000
	GO:0005976	polysaccharide metabolic process	26 (30.23%)	865 (5.71%)	0.000000	0.000000
	GO:0042546	cell wall biogenesis	14 (16.28%)	177 (1.17%)	0.000000	0.000000
	GO:0009832	plant-type cell wall biogenesis	10 (11.63%)	62 (0.41%)	0.000000	0.000000
	GO:0005975	carbohydrate metabolic process	31 (36.05%)	1493 (9.85%)	0.000000	0.000000
	GO:0071669	plant-type cell wall organization or biogenesis	14 (16.28%)	294 (1.94%)	0.000000	0.000000
	GO:0009698	phenylpropanoid metabolic process	11 (12.79%)	225 (1.48%)	0.000000	0.000003
	GO:0009699	phenylpropanoid biosynthetic process	10 (11.63%)	191 (1.26%)	0.000000	0.000006
	GO:0010413	glucuronoxylan metabolic process	4 (4.65%)	16 (0.11%)	0.000002	0.000075
	GO:0045229	external encapsulating structure organization	16 (18.6%)	720 (4.75%)	0.000002	0.000102
	GO:0044550	secondary metabolite biosynthetic process	10 (11.63%)	381 (2.51%)	0.000058	0.002254
	GO:0044085	cellular component biogenesis	17 (19.77%)	1087 (7.17%)	0.000109	0.003995
	GO:0010191	mucilage metabolic process	3 (3.49%)	19 (0.13%)	0.000160	0.005498
	GO:0019748	secondary metabolic process	12 (13.95%)	613 (4.04%)	0.000170	0.005520
	GO:0045488	pectin metabolic process	2 (2.33%)	7 (0.05%)	0.000656	0.020164
	GO:0071555	cell wall organization	9 (10.47%)	432 (2.85%)	0.000754	0.022019

S4-NC-vs-S4-FC	GO:0009867	jasmonic acid mediated signaling pathway	2 (2.33%)	9 (0.06%)	0.001116	0.029633
	GO:0071395	cellular response to jasmonic acid stimulus	2 (2.33%)	9 (0.06%)	0.001116	0.029633
	GO:0000160	phosphorelay signal transduction system	5 (5.81%)	145 (0.96%)	0.001399	0.035041
	GO:0010087	phloem or xylem histogenesis	4 (4.65%)	86 (0.57%)	0.001440	0.035041
	GO:0044042	glucan metabolic process	9 (10.47%)	481 (3.17%)	0.001597	0.037313
	GO:0009753	response to jasmonic acid	2 (2.33%)	12 (0.08%)	0.002024	0.045463
	GO:0044036	cell wall macromolecule metabolic process	28 (14.58%)	266 (1.75%)	0.000000	0.000000
	GO:0010383	cell wall polysaccharide metabolic process	25 (13.02%)	238 (1.57%)	0.000000	0.000000
	GO:0045491	xylan metabolic process	22 (11.46%)	210 (1.39%)	0.000000	0.000000
	GO:0010410	hemicellulose metabolic process	22 (11.46%)	211 (1.39%)	0.000000	0.000000
	GO:0071554	cell wall organization or biogenesis	35 (18.23%)	746 (4.92%)	0.000000	0.000000
	GO:0009698	phenylpropanoid metabolic process	17 (8.85%)	225 (1.48%)	0.000000	0.000001
	GO:0009699	phenylpropanoid biosynthetic process	14 (7.29%)	191 (1.26%)	0.000000	0.000016
	GO:0042546	cell wall biogenesis	13 (6.77%)	177 (1.17%)	0.000000	0.000039
	GO:0009808	lignin metabolic process	6 (3.13%)	26 (0.17%)	0.000001	0.000061
	GO:0005976	polysaccharide metabolic process	29 (15.1%)	865 (5.71%)	0.000001	0.000112
	GO:0009891	positive regulation of biosynthetic process	10 (5.21%)	115 (0.76%)	0.000002	0.000139
	GO:0009832	plant-type cell wall biogenesis	7 (3.65%)	62 (0.41%)	0.000013	0.000824
	GO:0005975	carbohydrate metabolic process	38 (19.79%)	1493 (9.85%)	0.000022	0.001284
	GO:0071669	plant-type cell wall organization or biogenesis	14 (7.29%)	294 (1.94%)	0.000023	0.001284
	GO:0015766	disaccharide transport	3 (1.56%)	8 (0.05%)	0.000107	0.005160
	GO:0015772	oligosaccharide transport	3 (1.56%)	8 (0.05%)	0.000107	0.005160
	GO:0072593	reactive oxygen species metabolic process	15 (7.81%)	391 (2.58%)	0.000140	0.006340
	GO:0050896	response to stimulus	102 (53.13%)	6084 (40.14%)	0.000170	0.007310
	GO:0006950	response to stress	68 (35.42%)	3632 (23.96%)	0.000220	0.008535

	GO:0042743	hydrogen peroxide metabolic process	14 (7.29%)	363 (2.39%)	0.000221	0.008535
	GO:0044085	cellular component biogenesis	28 (14.58%)	1087 (7.17%)	0.000253	0.009303
	GO:0016051	carbohydrate biosynthetic process	6 (3.13%)	72 (0.48%)	0.000298	0.010283
	GO:0009312	oligosaccharide biosynthetic process	3 (1.56%)	11 (0.07%)	0.000306	0.010283
	GO:0044550	secondary metabolite biosynthetic process	14 (7.29%)	381 (2.51%)	0.000361	0.011626
	GO:0002252	immune effector process	9 (4.69%)	179 (1.18%)	0.000464	0.014344
	GO:0010413	glucuronoxylan metabolic process	3 (1.56%)	16 (0.11%)	0.000992	0.029371
	GO:0048518	positive regulation of biological process	16 (8.33%)	523 (3.45%)	0.001027	0.029371
	GO:0009893	positive regulation of metabolic process	13 (6.77%)	378 (2.49%)	0.001077	0.029703
	GO:0006558	L-phenylalanine metabolic process	3 (1.56%)	17 (0.11%)	0.001194	0.030720
	GO:1902221	erythrose 4-phosphate/phosphoenolpyruvate family amino acid metabolic process	3 (1.56%)	17 (0.11%)	0.001194	0.030720
	GO:0006059	hexitol metabolic process	2 (1.04%)	5 (0.03%)	0.001557	0.036418
	GO:0019401	alditol biosynthetic process	2 (1.04%)	5 (0.03%)	0.001557	0.036418
	GO:0019406	hexitol biosynthetic process	2 (1.04%)	5 (0.03%)	0.001557	0.036418
	GO:0009620	response to fungus	13 (6.77%)	401 (2.65%)	0.001826	0.041462
	GO:0019748	secondary metabolic process	17 (8.85%)	613 (4.04%)	0.002066	0.045570
	GO:0044036	cell wall macromolecule metabolic process	27 (17.09%)	266 (1.75%)	0.000000	0.000000
	GO:0045491	xylan metabolic process	24 (15.19%)	210 (1.39%)	0.000000	0.000000
	GO:0010410	hemicellulose metabolic process	24 (15.19%)	211 (1.39%)	0.000000	0.000000
	GO:0010383	cell wall polysaccharide metabolic process	25 (15.82%)	238 (1.57%)	0.000000	0.000000
S8-NC-vs-S8-FC	GO:0009832	plant-type cell wall biogenesis	14 (8.86%)	62 (0.41%)	0.000000	0.000000
	GO:0042546	cell wall biogenesis	20 (12.66%)	177 (1.17%)	0.000000	0.000000
	GO:0071554	cell wall organization or biogenesis	36 (22.78%)	746 (4.92%)	0.000000	0.000000
	GO:0009698	phenylpropanoid metabolic process	21 (13.29%)	225 (1.48%)	0.000000	0.000000

GO:0009808	lignin metabolic process	10 (6.33%)	26 (0.17%)	0.000000	0.000000
GO:0009699	phenylpropanoid biosynthetic process	18 (11.39%)	191 (1.26%)	0.000000	0.000000
GO:0044550	secondary metabolite biosynthetic process	21 (13.29%)	381 (2.51%)	0.000000	0.000000
GO:0019748	secondary metabolic process	26 (16.46%)	613 (4.04%)	0.000000	0.000000
GO:0005976	polysaccharide metabolic process	31 (19.62%)	865 (5.71%)	0.000000	0.000000
GO:0071669	plant-type cell wall organization or biogenesis	17 (10.76%)	294 (1.94%)	0.000000	0.000001
GO:0010413	glucuronoxylan metabolic process	5 (3.16%)	16 (0.11%)	0.000000	0.000020
GO:0006558	L-phenylalanine metabolic process	5 (3.16%)	17 (0.11%)	0.000001	0.000025
GO:1902221	erythrose 4-phosphate/phosphoenolpyruvate family amino acid metabolic process	5 (3.16%)	17 (0.11%)	0.000001	0.000025
GO:0044085	cellular component biogenesis	28 (17.72%)	1087 (7.17%)	0.000007	0.000257
GO:0005975	carbohydrate metabolic process	33 (20.89%)	1493 (9.85%)	0.000024	0.000819
GO:0009891	positive regulation of biosynthetic process	8 (5.06%)	115 (0.76%)	0.000027	0.000879
GO:0009694	jasmonic acid metabolic process	10 (6.33%)	190 (1.25%)	0.000030	0.000946
GO:0045229	external encapsulating structure organization	20 (12.66%)	720 (4.75%)	0.000060	0.001787
GO:0010087	phloem or xylem histogenesis	6 (3.8%)	86 (0.57%)	0.000276	0.007845
GO:0019438	aromatic compound biosynthetic process	32 (20.25%)	1668 (11%)	0.000456	0.012414
GO:0008272	sulfate transport	3 (1.9%)	16 (0.11%)	0.000563	0.014170
GO:0072348	sulfur compound transport	3 (1.9%)	16 (0.11%)	0.000563	0.014170
GO:0006595	polyamine metabolic process	5 (3.16%)	67 (0.44%)	0.000663	0.016057
GO:0009889	regulation of biosynthetic process	21 (13.29%)	984 (6.49%)	0.001362	0.031812
GO:0044711	single-organism biosynthetic process	36 (22.78%)	2091 (13.8%)	0.001425	0.032134
GO:1901362	organic cyclic compound biosynthetic	33 (20.89%)	1892 (12.48%)	0.001896	0.041339

	process				
GO:0045488	pectin metabolic process	2 (1.27%)	7 (0.05%)	0.002191	0.046224
GO:0006950	response to stress	54 (34.18%)	3632 (23.96%)	0.002317	0.047348

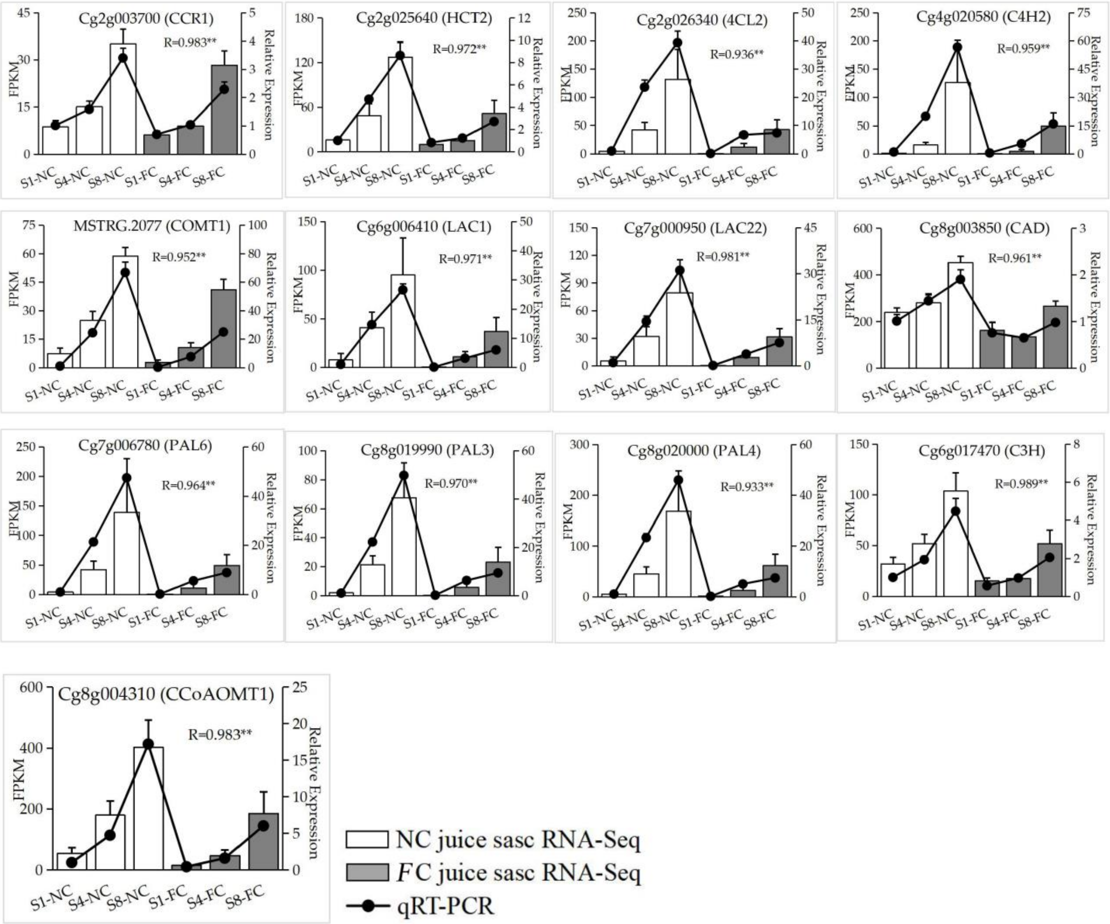

Supplementary Figure S3. Validation of expression patterns by qRT-PCR of selected DEGs identified in the RNA-seq analysis. The left Y-axis represents expression levels calculated by the fragments per kilobase per million reads (FPKM) method corresponding to the histogram plots. The right Y-axis represents the relative expression levels corresponding to the line plots. The corresponding bar plot shows the standard deviations (denoted as error bars). The relationship between the RNA-Seq and qRT-PCR in NC and FCjuice sacs was analyzed, respectively based on Pearson's correlation analysis and R represents correlation coefficients.
